# Insulin Resistance and Beta-Cell Function in the Context of Dyslipidemia in a Non-obese Indian Population With Type 2 Diabetes

**DOI:** 10.7759/cureus.91951

**Published:** 2025-09-10

**Authors:** Diptika Tiwari, Pramod Tripathi, Nidhi S Kadam, Baby Sharma, Anagha Vyawahare, Thejas Kathrikolly, Malhar Ganla, Banshi Saboo

**Affiliations:** 1 Department of Research, Freedom from Diabetes Research Foundation, Pune, IND; 2 Department of Endocrinology, Diabetes Care and Hormone Clinic, DiaCare, Ahmedabad, IND

**Keywords:** beta cell function, dyslipidemia, insulin resistance, non-obese, type 2 diabetes

## Abstract

Introduction

Insulin resistance (IR) and impaired beta-cell function (BCF) are central to type 2 diabetes (T2D) pathogenesis and influence lipid metabolism, causing dyslipidemia. While most dyslipidemia studies focus on obese individuals, data on non-obese populations are limited. This study explored the association between IR, BCF, and lipid profiles in non-obese Indian T2D patients.

Methods

A cross-sectional analysis was conducted on T2D patients (n=667) with a body mass index <25 kg/m², not on insulin or lipid-lowering medications, who participated in a one-year lifestyle intervention program at the Freedom from Diabetes Clinic, India. Clinical, anthropometric, and biochemical data were extracted from existing records. IR and BCF were assessed using homeostatic model assessment of IR (HOMA2-IR) and BCF (HOMA2-%B).

Results

The median age, HbA1c, and diabetes duration were 50 years, 7.6%, and nine years, respectively. Overall, 73.3% (n=489) were male, 4.2% (n=28) had IR (HOMA2-IR≥2.0), and 69.9% (n=466) had reduced BCF (HOMA2-%B<50). HOMA2-IR showed a positive correlation with triglycerides and non-high-density lipoprotein cholesterol (non-HDL-C) (P<0.001) and a negative correlation with HDL-C (P<0.001). HOMA2-%B was negatively associated with total cholesterol, low-density lipoprotein cholesterol (LDL-C), non-HDL-C, and HDL-C (P<0.05). Receiver operating characteristic (ROC) analysis demonstrated that triglycerides (area under the curve (AUC)=0.648, P=0.008), non-HDL-C (AUC=0.626, P=0.023), and HDL-C in men (AUC=0.706, P=0.003) had fair discriminatory ability for IR. HOMA2-%B failed to discriminate lipid abnormalities.

Conclusion

In non-obese Indian T2D patients, both IR and beta-cell dysfunction are associated with dyslipidemia. However, only IR demonstrated a fair discriminatory ability for identifying abnormal lipid profiles, whereas BCF did not. These findings underscore the need for further studies to understand the metabolic drivers of lipid abnormalities in this population.

## Introduction

Type 2 diabetes (T2D) is a heterogeneous group of chronic metabolic disorders that accounts for approximately 90% of all diagnosed diabetes cases worldwide [1]. It is commonly associated with insulin resistance (IR), obesity, dyslipidemia, and hypertension, leading to an increased risk of cardiovascular diseases [1,2]. While obesity is a dominant risk factor, a significant proportion of individuals with T2D, particularly in Asian populations, maintain a normal to low body mass index (BMI <23 kg/m²), highlighting a distinct pathophysiological phenotype [3,4]. Non-obese individuals with T2D exhibit metabolic disturbances in the absence of overt adiposity and account for up to 50% of T2D cases in several Asian populations [3,5].

The disease mechanism in non-obese patients with T2D may differ substantially from that in obese individuals [6]. While the interplay between IR and pancreatic beta-cell function (BCF) remains central to T2D pathogenesis [7], non-obese patients often demonstrate relatively mild IR but more pronounced BCF [8]. However, recent studies have highlighted that even in lean individuals, regional adipose distribution, particularly visceral fat, contributes significantly to IR [9]. IR, along with impaired BCF, disrupts lipid metabolism and may explain the dyslipidemic profiles often seen in T2D patients [10,11]. Mechanistically, IR can elevate triglyceride levels by impairing the suppression of lipolysis and increasing hepatic fatty acid influx, while concurrently reducing high-density lipoprotein cholesterol (HDL-C) levels by limiting ApoA-I expression, a key component in HDL-C synthesis [12,13]. Simultaneously, BCF-related insulin deficiency diminishes the inhibition of very low-density lipoprotein cholesterol (VLDL-C) secretion, lipoprotein lipase activation, and low-density lipoprotein cholesterol (LDL-C) receptor activity, further contributing to lipid abnormalities [14,15]. These interrelated mechanisms underscore the need to consider both IR and BCF when evaluating cardiometabolic risk in non-obese patients with T2D.

Although obesity-linked T2D is well characterized, there remains a paucity of data exploring how IR and BCF relate to dyslipidemia in non-obese individuals with T2D, particularly in South Asian populations [16]. Given this gap in the literature, the present cross-sectional study utilized retrospective data to explore the association between IR, BCF, and dyslipidemia in non-obese individuals with T2D. As an exploratory analysis, the goal was to generate preliminary insights rather than establish predictive markers, acknowledging the inherent limitations of cross-sectional designs in inferring causality. Despite these constraints, examining this relationship may offer valuable insights into disease pathophysiology and help identify potential targets for early intervention to mitigate cardiovascular risk in this distinct patient population.

## Materials and methods

Study design and population

This cross-sectional study was conducted at the Freedom from Diabetes Clinic in Pune, India. Retrospective data were extracted for 667 patients who enrolled in a one-year online diabetes management program. Participants were recruited in monthly batches, and all eligible individuals who joined between June 2021 and June 2023 were included. The inclusion criteria were as follows: T2D patients aged between 18 and 75 years, on oral hypoglycemic agents (OHAs), and without any significant diabetes-related complications such as nephropathy, or chronic illnesses including liver or renal disease, or other comorbidities such as heart disease. Pregnant and lactating women were also excluded from the study. Additionally, participants who were on exogenous insulin therapy at baseline were excluded, as exogenous insulin alters endogenous insulin dynamics and could confound the calculation of IR (homeostatic model assessment of IR (HOMA2-IR)) and BCF (HOMA2-%B), which were key metabolic outcomes in this study. Similarly, individuals receiving lipid-lowering medications at baseline were excluded to avoid potential confounding effects on IR and BCF, given the independent impact of these drugs on metabolic parameters.

Ethics approval and consent to participate

This study was approved by the Institutional Ethics Committee (Ref. No. FFDRF/IEC/2024/7) and registered in the Clinical Trials Registry of India (CTRI) (CTRI/2024/03/064596). Owing to the retrospective nature of the study, the requirement for informed consent was waived by the Ethics Committee. The extracted retrospective data were anonymized and handled with strict confidentiality to ensure the privacy of the patients included in the study. This study adhered to the ethical principles and was conducted in accordance with the Declaration of Helsinki and its latest amendments.

Measurement of anthropometric and biochemical parameters

Data from all eligible patients were extracted from the clinical database of the Freedom from Diabetes Clinic, Pune, India. The extracted data included anthropometry (height and weight), medical history (date of diabetes diagnosis, comorbidities, and medication status), and biochemical parameters such as HbA1c, fasting blood glucose (FBG), fasting insulin, and lipid profile. Non-HDL-C was calculated from the lipid profile using the following formula: $\text{non-HDL-C} = \text{total cholesterol (TC)} - \text{HDL-C}$

IR and BCF were assessed using the homeostatic model assessment (HOMA) calculator (https://www.rdm.ox.ac.uk/about/our-facilities-and-units/DTU/software/homa) for HOMA2-IR and HOMA2-%B, respectively [17]. IR was defined as HOMA2-IR ≥2, and reduced BCF was defined as HOMA2%B ≤50 [18,19].

Statistical analysis

Statistical analyses were performed using IBM SPSS Statistics for Windows, Version 21 (Released 2012; IBM Corp., Armonk, New York, United States). Owing to the skewed distribution of continuous variables, descriptive statistics are presented as medians (interquartile ranges). Categorical variables were reported as frequencies and percentages. Non-parametric tests (Mann-Whitney tests) were used to determine the significance of the differences in the medians between the groups. The chi-square test was used to evaluate the differences in proportions between groups for categorical variables. Partial correlation analysis was used to determine the relationship between HOMA2-IR, HOMA2-%B, and lipid profiles by adjusting for confounding variables (age, diabetes duration, and sex). Unlike correlation analysis, which measures only the strength and direction of the association, receiver operating characteristic (ROC) analysis assesses how well lipid markers differ between varying IR and BCF levels. Therefore, ROC analysis was used to determine the association and discriminatory ability of the lipid profile against IR (HOMA2-IR) and BCF (HOMA-2-%B). The area under the curve (AUC) values were interpreted using conventional thresholds commonly applied in diagnostic research, where values of 0.5 - 0.6 indicate failed discrimination, 0.6 - 0.7 poor, 0.7 - 0.8 fair, 0.8 - 0.9 good, and ≥0.9 excellent discriminatory ability [20]. Due to sex-specific cut-offs for HDL-C (males>40 mg/dL; females>50 mg/dL), all analyses related to HDL-C were performed separately for males and females. The level of statistical significance was set at P<0.05.

## Results

Sociodemographic and clinical characteristics of patients

The demographic and clinical characteristics of the patients are presented in Table 1. This study analyzed data from 667 patients with T2D, recruited from 125 cities across 24 states in India, thereby reflecting a geographically diverse population, all of whom had a BMI <25 kg/m² (non-obese). Of these, 73.3% (n=489) were male, the majority were married (92.7%, n=618), had an educational status of graduate or below (57.0%, n=380), and were salaried (employment) (72.1%, n=481). All enrolled patients were on OHAs but not insulin or lipid-lowering medications, and 19.6% (n=131) were on antihypertensive medication.

**Table 1:** Sociodemographic characteristics, glycemic, and lipid profile variation stratified by HOMA2-IR and HOMA2-%B status (N=667) Data are presented as frequencies and percentages or medians (interquartile range). Categorical variables were compared using the chi-square (χ2) test, and continuous variables were compared using the Mann-Whitney U test. BMI: body mass index; HbA1c: glycated hemoglobin; LDL-C: low-density lipoprotein cholesterol; HDL-C: high-density lipoprotein cholesterol; HOMA2-IR: homeostatic model assessment of insulin resistance; HOMA2-%B: homeostatic model assessment of beta-cell function; IR: insulin resistance; BCF: beta-cell function *Values when taking oral hypoglycemic agents. Diabetes duration was categorized based on the median value.

Parameters (N, %)		Total N=667	Chi-square test results							
			NO-IR (HOMA2-IR<2.0) N=639	IR (HOMA2-IR ≥ 2.0) N=28	χ2 /U value	P-value	Reduced BCF (HOMA2-%B≤50) N=466	Preserved BCF (HOMA2-%B>50) N=201	χ2 /U value	P-value
Age (years)	≤ 50 years	358 (53.7)	340 (95.0)	18 (5.0)	1.341	0.247	247 (69.0)	111 (31.0)	0.278	0.598
	> 50 years	309 (46.3)	299 (96.8)	10 (3.2)			219 (70.9)	90 (29.1)		
Sex	Male	489 (73.3)	471 (96.3)	18 (3.7)	1.206	0.272	349 (71.4)	140 (28.6)	1.972	0.160
	Female	178 (26.7)	168 (94.4)	10 (5.6)			117 (65.7)	61 (34.3)		
BMI (Kg/m^2^)	Normal (18.5– 22.9)	317 (47.5)	312 (98.4)	5 (1.6)	10.365	0.001	237 (74.8)	80 (25.2)	6.885	0.009
	Overweight (23–24.9)	350 (52.5)	327 (93.4)	23 (6.6)			229 (65.4)	121 (34.6)		
Family History	Yes	505 (75.7)	484 (95.8)	21 (4.2)	0.007	0.932	352 (69.7)	153 (30.3)	0.026	0.872
	No	162 (24.3)	155 (95.7)	7 (4.3)			114 (70.4)	48 (29.6)		
Diabetes Duration	< 10 years	356 (53.4)	339 (95.2)	17 (4.8)	0.645	0.422	236 (66.3)	120 (33.7)	4.630	0.031
	≥ 10 years	311 (46.6)	300 (96.5)	11 (3.5)			230 (74.0)	81 (26.0)		
Parameters, Median (IQR) Mann-Whitney U Test Result										
HbA1c (%)*		7.4 (6.6 – 9.0)	7.4 (6.5 – 8.9)	7.5 (6.8 – 10.3)	7445.0	0.136	7.8 (6.7 – 9.4)	6.8 (6.2 – 7.8)	30387.00	<0.001
Fasting Blood Glucose (mg/dL) *		126.7 (106.0 – 153.3)	126.0 (105.8 – 152.6)	143.5 (121.8 – 179.1)	6012.50	0.003	140.0 (118.1 – 174.7)	103.0 (92.0 – 115.6)	14536.00	<0.001
Fasting Insulin (µU/ml)		5.8 (3.7 – 9.2)	5.6 (3.6 – 8.7)	18.6 (17.2 – 27.9)	502.50	<0.001	4.8 (3.1 – 7.0)	9.3 (6.2 – 12.8)	19026.50	<0.001
Total Cholesterol (mg/dL)		172 (155.0 – 196.0)	172.0 (155.0 – 195.2)	173.0 (167.2 – 202.7)	7439.50	0.134	174.0 (156.1 –198.0)	170.0 (152.1 – 187.8)	42077.50	0.037
Triglycerides (mg/dL)		114 (87.0 – 154)	112.5 (86.0 – 152.0)	148.0 (109.0 – 205.2)	6285.50	0.008	111.0 (85.0 – 155.0)	121 (91.5 – 154.0)	43939.00	0.205
LDL-C (mg/dL)		110 (94 – 129)	109.5 (93.3 – 121.9)	120.0 (100.5 –130.9)	7025.50	0.056	110.0 (94.0 –132.0)	108.0 (95.0 –132.2)	42544.50	0.060
Non-HDL-C (mg/dL)		130.0 (114.0 – 152.0)	130.0 (113.0 – 151.3)	141.0 (125.0 – 169.7)	6674.50	0.023	131.0 (114.0 – 154.0)	129.0 (113.6 – 147.0)	43472.00	0.141
HDL-C (mg/dL)	Male (n=489)	41.0 (36.0 – 46.0)	41.0 (36.0 – 46.0)	36.0 (31.7 – 40.7)	2489.50	0.003	41.0 (36.0 – 47.0)	38.5 (35.0 – 45.0)	20848.00	0.011
	Female (n=178)	45.0 (39.0 – 52.0)	45.5 (39.0 – 52.0)	39.5 (37.7 – 50.0)	621.50	0.167	46.0 (39.2 – 54.0)	44.0 (39.0 – 50.9)	3264.50	0.351

Baseline sociodemographic and clinical data revealed that the median age, weight, BMI, and diabetes duration were 50 years (IQR, 43-58 years), 64 kg (IQR, 58.7-69 kg), 23.0 kg/m² (IQR, 21.8-24.1 kg/m^2^), and nine years (IQR, 5.4-14.2 years), respectively. Regarding glycemic parameters, 58.8% (n=392) of the patients showed poor glycemic control (HbA1c ≥ 7%), 81.3% (n=542) had elevated FBG levels (≥ 100 mg/dL), 4.2% (n=28) showed IR (HOMA2-IR ≥ 2.0), and 69.8% (n=639) showed reduced BCF (HOMA2-%B≤50). The median HOMA2-IR and HOMA2-%B were 0.73 (IQR, 0.45-1.14) and 35.8 (IQR, 22.5-53.6), respectively. A significantly higher proportion of patients without IR had a normal BMI (P=0.001). Preserved BCF was more commonly observed in the overweight category (P=0.009), while reduced BCF was significantly associated with a diabetes duration of more than 10 years (P=0.031).

Furthermore, 11.4% (n=76) of patients had hypertension, defined as a blood pressure ≥ 140/90 mm/Hg. Additionally, the prevalence of isolated single-parameter dyslipidemia, as determined by the standard cut-offs [21], was highest for HDL-C (≤ 50 mg/dL) in females (69.7%, n=124), followed by LDL-C (≥ 100 mg/dL) (65.1%, n=434) and HDL-C (≤ 40 mg/dL) in males (48.9%, n=239). The prevalence of high TC (≥ 200 mg/dL), triglycerides (≥ 150 mg/dL), and non-HDL-C (≥ 130 mg/dL) levels was 19.9% (n=133), 27.0% (n=180), and 49.6% (n=331), respectively.

Lipid profile stratified by insulin resistance (HOMA2-IR) and beta-cell function (HOMA2-%B)

Table 1 presents the lipid profiles of the study cohort, categorized by the presence and absence of IR (HOMA2-IR ≥ 2.0 vs. <2.0) and preserved vs. reduced BCF (HOMA2-%B >50 vs. ≤50). Notably, none of the participants (n=667) were on medications for dyslipidemia at the time of assessment. Between the HOMA2-IR groups, significant differences were observed in triglycerides, non-HDL-C, and HDL-C (in males) (P<0.05). In contrast, statistically marginally significant differences were found in LDL-C levels (P=0.056), and no statistically significant differences were observed in TC and HDL-C levels (in females) (P>0.1). The IR group (HOMA2-IR ≥ 2.0) exhibited elevated median levels of triglycerides, non-HDL-C, and LDL-C, along with reduced HDL-C levels in both males and females; however, median TC values remained within the optimal reference range across both groups.

Subsequently, significant differences were observed between the HOMA2-%B groups for TC and HDL-C (in males) (P<0.05). Other lipid profiles, including triglycerides, LDL-C, non-HDL-C, and HDL-C (in females), showed no significant differences (P>0.05). The preserved BCF group (HOMA2-%B>50) showed lower median values for TC, LDL-C, non-HDL-C, and HDL-C and higher median values for triglyceride levels compared to the reduced BCF group (HOMA2-%B≤50) (Table 1).

Correlation of insulin resistance (HOMA2-IR) and beta-cell function (HOMA2-%B) with lipid parameters

Table 2 presents the results of a partial correlation analysis performed to assess the relationship between lipid profiles and HOMA2-IR and HOMA2-%B. This analysis was controlled for potential confounding variables, including age, sex, disease duration, and BMI. The analysis revealed that HOMA2-IR was significantly positively correlated with TC (P=0.038), triglycerides (P<0.001), and non-HDL-C (P<0.001), whereas it was significantly negatively correlated with HDL-C (P<0.001). For HOMA2-%B, a significant negative correlation was observed with TC (P=0.003), LDL-C (P=0.021), non-HDL-C (P=0.030), and HDL-C (P=0.002). No significant correlation was observed between LDL-C and HOMA2-IR or between triglyceride levels and HOMA-2%B. Although the correlations were weak, the findings were statistically significant, underscoring the interaction between lipid metabolism and IR, with particular emphasis on the roles of triglycerides and HDL-C.

**Table 2:** Partial correlation between lipid profile, HOMA2-IR, and HOMA2-%B HOMA2-IR: homeostatic model assessments of insulin resistance; HOMA2-%B: homeostatic model assessments of beta-cell function; LDL-C: low-density lipoprotein cholesterol; HDL-C: high-density lipoprotein cholesterol

Parameter (N=667)	HOMA2-IR		HOMA2%-B	
	R (correlation coefficient)	P-value	R (correlation coefficient)	P-value
Total Cholesterol	0.081	0.038	-0.116	0.003
Triglycerides	0.265	<0.001	-0.026	0.501
LDL-C	0.060	0.122	-0.089	0.021
Non-HDL-C	0.146	<0.001	-0.084	0.030
HDL-C	-0.192	<0.001	-0.120	0.002

Receiver operating characteristic (ROC) curve analysis for lipid profile, insulin resistance, and beta-cell function

**Figure 1:**
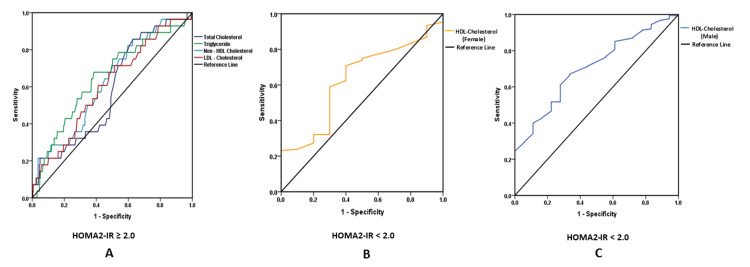
Receiver operating characteristic (ROC) curve showing the association of lipid profiles with insulin resistance in non-obese type 2 diabetes A: ROC analysis of total cholesterol, triglycerides, non-high-density lipoprotein cholesterol (non-HDL-C), and low-density lipoprotein cholesterol (LDL-C) with homeostatic model assessment of insulin resistance (HOMA2-IR) in the overall study cohort; B: ROC analysis of high-density lipoprotein cholesterol (HDL-C) with homeostatic model assessment of insulin resistance (HOMA2-IR) in females; C: ROC analysis of high-density lipoprotein cholesterol (HDL-C) with homeostatic model assessment of insulin resistance (HOMA2-IR) in males.

**Figure 2:**
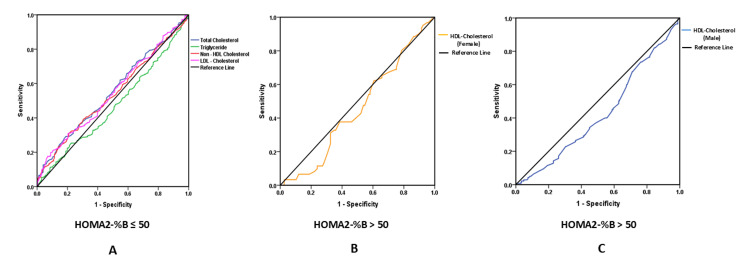
Receiver operating characteristic (ROC) curve showing the association of lipid profiles with beta-cell function in non-obese type 2 diabetes A: ROC analysis of total cholesterol, triglycerides, non-high-density lipoprotein cholesterol (non-HDL-C), and low-density lipoprotein cholesterol (LDL-C) with homeostatic model assessment of beta-cell function (HOMA2-%B) in the overall study cohort; B: ROC analysis of high-density lipoprotein cholesterol (HDL-C) with homeostatic model assessment of beta-cell function (HOMA2-%B) in females; C: ROC analysis of high-density lipoprotein cholesterol (HDL-C) with homeostatic model assessment of beta-cell function (HOMA2-%B) in males.

ROC curve analysis was performed to explore the ability of lipid parameters to distinguish between patients with and without IR (HOMA2-IR) (Figure 1) and reduced and preserved BCF (HOMA2-%B) (Figure 2). Given the low proportion of IR cases (4%), the findings were interpreted with caution, and confidence intervals were reported to account for potential instability in AUC estimates.

Table 3 presents the AUC comparisons for HOMA2-IR and HOMA2-%B across various lipid parameters. HOMA2-IR demonstrated discriminatory ability for identifying lipid abnormalities, with significant associations observed for triglycerides (AUC=0.648, P=0.008) and non-HDL-C (AUC=0.626, P=0.023). LDL-C showed marginal significance (AUC=0.607, P=0.056), while TC failed to show discriminatory ability (AUC=0.584, P=0.134). HDL-C displayed a strong sex-specific pattern, with a significant association in males (AUC=0.706, P=0.003), but a weaker, non-significant association in females (AUC=0.630, P=0.168) (Table 3). In contrast, HOMA2-%B failed to show any discriminatory ability for lipid abnormalities (TC (AUC=0.511, P=0.037), triglyceride (AUC=0.469, P=0.205), LDL-C (AUC=0.546, P=0.060), non-HDL-C (AUC=0.536, P=0.141), HDL-C (males) (AUC=0.427, P=0.011), and HDL-C (AUC=0.457, P=0.351)) (Table 3). Overall, the AUC findings suggest that IR is moderately associated with an altered lipid profile, particularly among males, as reflected by HDL-C levels (Figure 1). In contrast, BCF did not demonstrate any discriminatory ability for identifying dyslipidemia in this population (Figure 2).

**Table 3:** AUC Comparison for HOMA2-IR and HOMA2-%B in lipid profile prediction AUC: area under the curve; CI: confidence interval; HOMA2-IR: homeostatic model assessments of insulin resistance; HOMA2-%B: homeostatic model assessments of beta-cell function; LDL-C: low-density lipoprotein cholesterol; HDL-C: high-density lipoprotein cholesterol

Parameter (N=667)		HOMA2-IR				HOMA2%-B			
		AUC	95% CI		P-value	AUC	95% CI		P-value
			Lower bound	Upper bound			Lower bound	Upper bound	
Total Cholesterol		0.584	0.485	0.682	0.134	0.511	0.505	0.597	0.037
Triglycerides		0.648	0.540	0.756	0.008	0.469	0.422	0.516	0.205
LDL-C		0.607	0.502	0.712	0.056	0.546	0.499	0.592	0.060
Non-HDL-C		0.626	0.525	0.728	0.023	0.536	0.490	0.582	0.141
HDL-C	Male	0.706	0.600	0.812	0.003	0.427	0.372	0.482	0.011
	Female	0.630	0.618	0.792	0.168	0.457	0.371	0.544	0.351

## Discussion

This study provides valuable insights into the relationship between IR, BCF, and lipid profiles in Indian patients with T2D without obesity. Both IR and BCF appear to influence lipid metabolism; however, IR demonstrated a stronger association with abnormal lipid parameters. Specifically, elevated triglycerides, non-HDL-C, and reduced HDL-C levels in males were significantly associated with HOMA2-IR, highlighting its relevance in identifying patients at increased cardiometabolic risk. In contrast, BCF showed limited association with dyslipidemia and no discriminatory utility. Notably, preserved BCF (HOMA2-%B >50) was negatively associated with HDL-C levels in male patients.

The prevalence of dyslipidemia was notable, particularly for HDL-C (69.7%, n=124 in females and 48.9%, n=239 in males), followed by LDL-C (65.1%, n=434) and non-HDL-C (49.6%, n=331). These findings align with previous Indian studies reporting that dyslipidemia is a common feature in patients with T2D, irrespective of their obesity status [22,23]. Dyslipidemia in our cohort may be partially attributed to IR and impaired BCF, which are key metabolic disturbances that adversely affect lipid metabolism and contribute to cardiovascular risk, particularly in non-obese T2D patients [11,24]. Therefore, isolating the effects of IR and BCF on lipid metabolism in non-obese patients is critical for planning better treatment strategies.

Our study found optimal IR levels (measured as HOMA2-IR (0.73 (IQR, 0.45-1.14))) in non-obese T2D patients, while revealing low BCF, measured as HOMA2-%B (35.8 (IQR, 22.5-53.6)). These findings align with those of Mahmoud et al. (2021) [8], who reported that non-obese T2D patients exhibited lower IR and reduced BCF than their obese counterparts in the Egyptian population. While IR is often associated with obesity in T2D, our study showed that 4.2% (n=28) of non-obese patients had elevated HOMA2-IR values (≥ 2.0). This observation aligns with previous reports in Asian populations, reinforcing the idea that IR can occur without obesity in T2D patients, highlighting its importance beyond traditional obesity in non-obese T2D patients [9,25].

The relationship between IR, BCF, and dyslipidemia in T2D is complex, with each factor influencing the other. The differences observed in lipid profiles based on IR and BCF provide key insights into lipid alterations in non-obese T2D patients. Similar to other studies, our results showed that higher HOMA2-IR (≥ 2.0) was significantly correlated with elevated triglycerides, non-HDL-C, and decreased HDL-C (in males) (P<0.05), suggesting that IR might play an important role in the dysregulation of lipid metabolism in non-obese T2D patients [11,26]. Although our study corroborates the pivotal role of IR in lipid metabolism dysregulation among non-obese T2D patients, the underlying mechanisms remain to be fully elucidated. It has been hypothesized that IR in non-obese individuals may disproportionately affect hepatic lipid metabolism, leading to increased VLDL production and subsequent dyslipidemia, consistent with previous findings [27]. However, no previous study has reported an association between IR and dysregulated lipid profiles in non-obese T2D patients.

BCF plays a central role in lipid metabolism via insulin-mediated pathways. Insulin suppresses adipose tissue lipolysis, reducing circulating free fatty acids, inhibiting hepatic VLDL-C production, activating lipoprotein lipase to promote triglyceride clearance, and enhancing LDL-C receptor expression for cholesterol uptake [14,28]. Early beta-cell stress may initially lead to compensatory hyperinsulinemia, but over time, it may transition to insulin secretory failure. This progression is associated with increased hepatic lipogenesis, reduced clearance of circulating lipids, and dysregulated adipose tissue lipid turnover, all of which contribute to dyslipidemia and heightened cardiovascular risk [15,28]. This framework supports the role of deregulated BCF in lipid metabolism abnormalities. However, in our study cohort, BCF measured using HOMA2-%B showed a limited role in dyslipidemia. While HOMA2-%B did not show significant differences with most lipid parameters, notable exceptions occurred in TC and HDL-C levels (in males). This sex-specific finding could be attributed to the high proportion of males and hormonal differences, as estrogen in females plays a protective role in HDL metabolism, potentially buffering the effects of beta-cell dysfunction on lipid levels [29]. Another explanation could be that beta-cell dysfunction does not directly impair HDL-C metabolism and could reflect preserved insulin sensitivity despite reduced insulin secretion [13]. The lack of association between HOMA2-%B and dyslipidemia aligns with previous findings in T2D patients, irrespective of obesity status [10,27]. Similar patterns were observed in the AUC analysis, where higher triglyceride levels and lower HDL-C levels (in males) showed discriminatory power for IR and no IR, respectively, while none of the lipid parameters showed discriminatory power for BCF. This suggests that beta-cell dysfunction may have a limited role in dyslipidemia development in non-obese T2D patients, which may be attributed to the fact that the primary driver of dyslipidemia is IR rather than impaired insulin secretion [30].

In this exploratory study, most associations observed, though statistically significant, showed poor-to-fair discriminatory performance based on AUC values. These findings are not intended for immediate clinical application but offer preliminary insights into metabolic interactions in this distinct non-obese T2D population. The modest effect sizes highlight the need to distinguish statistical significance from clinical relevance, especially within a cross-sectional framework. Nevertheless, the consistent associations between HOMA2-IR and lipid parameters provide meaningful insight into early metabolic alterations and emphasize the distinct roles of IR and beta-cell dysfunction in this phenotype.

Limitations

While this study provides valuable insights into IR, BCF, and dyslipidemia in non-obese T2D patients, it is important to acknowledge its limitations. First, the cross-sectional design of the study precludes the establishment of a causal relationship between IR, BCF, and dyslipidemia. A reverse causal relationship may exist between lipid profiles, IR, and BCF. Longitudinal studies are necessary to confirm the temporal relationship between these variables and determine whether changes in IR or BCF lead to dyslipidemia in non-obese T2D patients over time. Secondly, the data were extracted from a clinical database, which may have introduced a selection bias concerning data completeness, potentially influencing the study’s findings. Additionally, for the non-obese criteria, we included patients with a BMI of less than 25 kg/m² (as per the Asian Pacific guideline by WHO) [4], which includes both normal-weight and overweight individuals, thereby introducing variability that may confound the association between IR, BCF, and dyslipidemia. Moreover, the lack of information on confounding factors such as diet, physical activity, and medication use (OHAs) could significantly affect IR, limiting the study’s interpretability. Furthermore, a key finding of our study was the association between IR and lipid abnormalities; however, this was derived from a relatively small subgroup of patients with IR (n=28) compared to the larger group with no IR (n=639). While this imbalance may limit the statistical power for subgroup comparisons, the comparisons between the IR and non-IR groups were performed primarily for descriptive purposes, and all analyses were conducted cautiously to minimize bias and ensure the robustness of the interpretation. Importantly, the consistency of the observed trends despite the small high-IR subgroup suggests a biologically relevant association that warrants confirmation in larger, more evenly distributed cohorts. Finally, the single-center nature of the study may limit the generalizability of the findings to a broader Indian population with different ethnicities. However, despite being a single-center study, it includes patients from 24 states and 125 cities, which enhances the diversity of the cohort and reduces location-specific bias. Additionally, the eligibility criteria of data completeness further strengthened the validity of the subgroup analyses, especially in small high-IR subgroups, by minimizing missing data bias and ensuring consistent analysis. Despite these limitations, this cross-sectional study offers meaningful and generalizable insights, adding valuable evidence to the field. These findings can serve as a foundation for future multicenter longitudinal studies to further validate the observed trends.

## Conclusions

In conclusion, this study suggests that both IR and BCF are linked to altered lipid profiles in non-obese Indian patients with T2D. However, IR showed stronger associations and fair discriminatory ability, particularly for triglyceride and HDL-C levels, indicating a potential role in lipid metabolism. In contrast, BCF showed limited association and no utility in identifying dyslipidemia. These exploratory, cross-sectional findings warrant cautious interpretation and highlight the need for longitudinal studies to clarify the metabolic mechanisms underlying dyslipidemia in this population.
